# The impact of the traditional male role norms on the posttraumatic stress disorder among Polish male firefighters

**DOI:** 10.1371/journal.pone.0259025

**Published:** 2021-10-27

**Authors:** Tomasz Daniel Jakubowski, Magdalena Maja Sitko-Dominik

**Affiliations:** Faculty of Social Sciences, Institute of Psychology, University of Silesia in Katowice, Katowice, Poland; Medical University of Vienna, AUSTRIA

## Abstract

**Background:**

The aim of this study was to explore potential associations between compliance with the traditional male role norms and posttraumatic stress disorder (PTSD) symptoms.

**Methods:**

The study was conducted on 135 male Polish firefighters. The study used the Impact Event Scale-Revised, the Relations/Social Support Scale and the Male Role Norms Scale.

**Results:**

The study revealed that the firefighters that suffered from probable PTSD (over 34% of all the respondents) seemed to comply with the male role norms more strictly than those without PTSD. The reverse was found to be true for the perceived social support. Social status norms and toughness norms understood as expectations that men should achieve high status by means of successful career, etc., and be tough and resilient at all costs to be perceived as “manly” were found to be positively associated with the development of PTSD, while in the case of perceived social support, a negative association was confirmed. The results might yield important clinical implications—traditional male role norms pertaining to toughness and social status might be associated with the increase in chances of developing PTSD after the exposure to potentially traumatic events.

**Conclusions:**

Firefighting as a stereotypically masculine occupation may be associated with the reinforcement of stereotypically masculine behaviors, which in turn is associated with a decreased ability to cope with potentially traumatic stimuli and favoring maladaptive behaviors. The results might suggest that addressing the beliefs about masculinity during psychological intervention in the case of PTSD might be beneficial especially among such masculinized groups as firefighters. The main limitations of the study are: participation of those more eager to participate and reveal personal information; the recall and report bias; the relatively small sample size; sociodemographic data omissions; the study group almost exclusively consisting of firefighters from large urban centers.

## Introduction

Firefighters are first responders, together with police officers, paramedics and others [1], who in their daily work are exposed to potentially traumatic events, which puts them at elevated risk of developing symptoms of posttraumatic stress disorder (PTSD) or even developing the full diagnosis [2]. The available data show that the rates of PTSD among firefighters are between 0% and 57% [3–11].

Among risk factors against PTSD among firefighters, there are maladaptative coping styles [11–15], alienation, feelings of insecurity and lack of personal control [16], high neuroticism, and fear of dying on duty [17], anxiety sensitivity [18,19], negative emotionality [20], burnout [21,22], length of service and lower rank [10,14,23–25], previous high exposure to traumatic events and previous psychopathology [11,26–29], elevated startle response [30], increased tendency to catastrophize [31], high hostility and low self-efficacy [3], negative self-appraisals [14,32], occupational stress, lack of social support and shift work [10,33–35], external locus of control in the workplace, low education and low resilience [24,36], and high social introversion and masculinity [37]. These risk factors seem to fall into three broad categories—intrapersonal [11–22,27–31,36], workplace-related [10,11,14,23–26] and social [10,16,33–35].

Another factor that may possibly contribute to the development of PTSD symptomatology in the face of adverse/traumatic situations might be the social expectations pertaining to masculinity and the way one relates to them at cognitive, emotional and behavioral levels [38]. Here the focus would be laid on maladaptive impact of trying to live up to the standard of the internalized masculine gender schema on the functioning of males, conceptualized by Pleck [38], where social expectations about masculinity (such as being tough, invulnerable, achieving high social status and not possessing any features considered as stereotypically feminine) might reinforce such behaviors and cognitions that limit the perceived and actual social support. This concept was further developed by O’Neil [39] in gender role conflict theory, which further posits that the strain put on a person’s well-being might be the result of fulfilling contradictory expectations of the masculine gender role, such as self-presenting as tough and invulnerable, even in adverse, resource expensive situations related to the expectation of achieving and maintaining high status [39]. Multiple studies have found an adverse effect of strict conforming to the male role norms on mental health care seeking as well as on mental health itself [40–46]. According to the Model of Masculinity and Men’s psychopathology [47], conforming to the schemas of masculinity reinforces maladaptive strategies of emotion regulation, which in turn may result in psychopathology in the longterm. Such norms might negatively impact the use of adaptive coping strategies and the perception of available social support, and diminish help-seeking behaviors perceived as showing “weakness” and thus, unmanly [38,39,47].

Currently, several studies have reported the existence of protective factors against PTSD among firefighters, such as training (and the associated ability to proficiently act in a stressfull and time-constricted situation) [48], task- and emotion-focused coping [12], emotional intelligence [49], a sense of humor (and the associated ability to give more adaptative meaning to any given situation) [26], social support [23,33,50–52], a sense of belonging (and the associated sense of connectedness, important in the face of adverse situations) [51], personal mastery, mindfulness, and conscientiousness [50,52–54]. These studies seem to divide coping resources into two broad categories: intra- [12,26,48–50,52–54] and inter-personal functioning [23,33,50–52].

As proved in previous studies [10,23,33–35,50–52,55], and the systematic review conducted by Geuzinge and associates [56], social support seems to play a significant role in processing trauma and coping with other mental health conditions. In the case of firefighters, the support obtained from family, friends, co-workers and in particular from the supervisors [55] seems to be especially beneficial in the context of traumatic experiences. It may promote and reinforce more adaptive coping strategies and attenuate PTSD symptomatology [10,23,33–35,50–52,55,56].

The first aim of the study is to evaluate potential associations between compliance with masculinity norms, social support, sociodemographic data and PTSD symptomatology. The second aim of the study is to evaluate potential differences in social support and compliance with masculinity norms between firefighters who meet the criteria for PTSD and those who do not. The third aim is to assess potential predictive values of male role norms, sociodemographic data and social support for PTSD symptomatology. The inclusion of sociodemographic data in the study is dictated by the fact that this is, to the authors’ knowledge, the only study of Polish firefighters. In such a case, the authors deemed valuable the association of the sociodemographic characteristics with the studied constructs.

Based on the above-mentioned data the following hypotheses were formulated:

- traditional male norms (anti-femininity, toughness and social status norms) increase the chances of developing PTSD symptoms among firefighters;
- social relations/support (perceived social support) decrease the chances of developing PTSD symptoms among firefighters.

## Materials and methods

### Participants and procedures

The current study is a part of the larger project pertaining to PTSD among first responders (firefighters, police officers and paramedics). The protocol of the current study—[doi.org/10.17504/protocols.io.bsd4na8w] [PROTOCOL DOI].

The study was conducted on 135 male Polish professional firefighters in active service—firefighting and taking part in rescue operations after traffic accidents. The participants were selected by means of the chain referral method [57,58] and convenience sampling [57]. The criterion for participating in the research was to be an active duty professional firefighter. In each case, the researchers turned to the commandants of the District (Municipal) Headquarters of the State Fire Service for permission to conduct the research. Having been granted permission, the researchers informed about the aim of the research; they were told that participation in the research was voluntary and anonymous and that the collected data would be used solely for academic purposes. All of them verbally consented to participate in the study. Each participant was presented with a set of three questionnaires and sociodemographic questions in an envelope. Further on, the participants were asked if they know anyone who might also want to participate.

The research was conducted by means of the traditional paper-and-pencil method in their workplace. They did not receive any payment for the participation in the research. The Academic Ethics Committee in the University of Silesia in Katowice granted the authors of this paper the permission to conduct the study (number 13/2019) as well as the permission to conduct it outside of the institution in the field sites. The research was conducted from September 2019 to December 2019 and involved firefighters from two districts, Śląskie and Łódzkie. Two hundred firefighters were invited to participate in the study. Out of 200 sets of questionnaires, 136 were returned to the authors of this paper. One female firefighter was excluded from the analysis. The basic sociodemographic characteristics are presented in Table 1.

**Table 1:** Sociodemographic characteristics of the sample.

Variables	n (%) or M(SD)
**Age** (10 omissions, n = 125)	35.38 (7.12)
**Education** (no omissions, n = 135)	
Primary/Vocational	4 (2.96%)
Secondary	83 (61.48%)
Higher	48 (35.56%)
**Length of service** (3 omissions, n = 132)	13.01 (7.03)
**Relationship** (1 omission, n = 134)	
Single	20 (14.93%)
Informal relationship	28 (20.90%)
Married	66 (49.25%)
Divorced	20 (14.93%)
**Relationship length** (41 omissions, n = 94)	8.42 (6.50)

The participants were asked to provide the following sociodemographic data: gender, age, marital status, length of the relationship, education and length of service. Besides, they were asked to tick off what they experienced during the service: experience of life threat/assault, seeing dead bodies/ death of a fellow firefighter, being injured. Next, they were asked how often they experienced such situations (from “*there were no such events” to “more than a dozen times”)*. This list constitutes an adaptation of the list used in the study conducted by Dudek [59].

### Measures

In the current study, dependent variables were measured with the Impact Event Scale-Revised (IES-R), while independent variables were measured with the Relations/Social Support Scale, the Male Role Norms Scale.

#### The Impact of Event Scale-Revised

The Polish adaptation of Daniel Weiss & Charles Marmar’s [60] IES-R by Juczyński & Ogińska-Bulik [61] was prepared in accordance with the rules of transcultural adaptation. It consists of 22 items scored on a 5-point Likert scale (from 0—“*definitely not*” to 4—“*definitely yes*”), in 3 sub-scales—intrusion (e.g., “When I recalled the event, the emotions returned”), hyperarousal (e.g., “Recalling this event made me sweat and I had breathing problems, dizziness, and my heart fluttered”) and avoidance (e.g., “I tried to avoid talking about the event”). The reliability of the whole scale in the current study was α = .96; the sub-scale reliability was α = .93, α = .89 and α = .88 for intrusions, hyperarousal and avoidance, respectively. The PTSD diagnosis is possible when the average result of the whole scale is higher than 1.5, which is indicated e.g. by Creamer et al. [62]. However Juczyński & Ogińska-Bulik [61] suggest a more restrictive approach: they suspect that the individual meets the criteria for the PTSD diagnosis, when his/her results in each of the scales exceed 1.5. The selected method of computing probable PTSD on the basis of IES-R scores involves the possession of mean values of all the three sub-scales equal to or higher than 1.5. This method seems to help decrease the chance of overestimating the prevalence of probable PTSD among the participants [61].

#### The Relations/Social Support Scale

The Scale by Skarżyńska [63] consists of 6 items (e.g., “I am surrounded by many people who are close to me”; “In important matters I can count on my friends’ help”) scored on a 5-point Likert scale (from “*I definitely don’t agree*” to “*I definitely agree*”). The reliability in the current study was α = .72.

#### The Male Role Norms Scale

*The Male Role Norms Scale* by Thompson & Pleck [64] was translated into Polish by the authors of this paper. The measure consists of 26 items scored on a 7-point Likert scale (from “*I definitely don’t agree*” to “*I definitely agree*”), in 3 sub-scales: social status (e.g., “Success at work must be the main aim in the man’s life”), toughness (e.g., “I like men who are totally self-confident”) and anti-femininity norms (e.g., “If I learnt about a man that is a hairdresser or a cook, I would wonder how masculine he is”). The reliability of the scale in the current study was α = .89, while the reliability of the sub-scales (social status, toughness and anti-femininity norms) was α = .86, α = .73 and α = .80, respectively.

### Statistical analysis

The sociodemographic data which were missing in the cases included in the statistical analysis were left unchanged. The researchers took into consideration only the information provided by the participants. The study included intrusions, hyperarousal, avoidance and the IES-R total score as dependent variables and social relations/support, social status norms, toughness norms, anti-femininity norms, age, education, relationship length, length of service, number of types of events, frequency of events as independent variables. The analytics software package Statistica version 13.1. [65] was used to compute descriptive statistics, correlations, intergroup differences (one-way ANOVA and the Mann-Whitney U test), univariate regression, backward stepwise multivariate regression and logistic regression.

To assess potential associations between compliance with masculinity norms, social support and PTSD symptomatology, due to non-normal distribution of the majority of variables (social relations/support, intrusions, hyperarousal, avoidance, the IES-R total score, toughness norms, anti-femininity norms, age, relationship length, number of types of events, frequency of events), Spearman’s rank correlation was utilized; and for the remainder (social status norms and length of service), Pearson’s correlation was used. The Mann–Whitney U test was utilized to evaluate potential differences in social relations/support and in compliance with toughness and anti-femininity norms between firefighters who meet the criteria for PTSD (the positive screen for the PTSD group) and those who do not (the negative screen for the PTSD group); while the one-way ANOVA was used in the case of social status norms. Different statistical methods were utilized in the case of the above mentioned due to non-normal distribution of the majority of the variables (except for social status norms), as suggested by Hollander, Wolfe & Chicken [66]. The assessment of potential predictors of PTSD symptomatology (male role norms, social support and sociodemographic data) was conducted by means of univariate regression; the potential model of predictors of PTSD symptomatology and their relative importance were assessed by means of multivariate stepwise backward regression; while the odds of developing PTSD were measured by means of logistic regression. The statistical significance was set on p< .05.

## Results

The basic descriptive statistics (mean and standard deviation) for relations/social support, intrusions, hyperarousal, avoidance, the IES-R total score, social status norms, toughness norms, anti-femininity norms, age, relationship length, length of service, number of types of events and frequency of events are summarized in Table 2.

**Table 2:** Descriptive statistics.

Variables	M	SD	Skew.	Kurt.
**Social relations/support**	3.37	0.78	0.59	-0.65
**Intrusions**	1.43	1.09	0.27	-1.28
**Hyperarousal**	1.44	0.99	0.19	-1.13
**Avoidance**	1.59	1.02	-0.03	-1.03
**The IES-R total score**	1.48	0.99	0.16	-1.23
**Social status norms**	4.40	0.99	0.20	0.35
**Toughness norms**	4.11	0.89	0.67	1.58
**Anti-femininity norms**	3.68	1.14	-0.30	-0.15
**Age**	35.9	6.63	0.31	-0.83
**Relationship length (yrs)**	8.20	6.41	0.92	-0.22
**Length of service (yrs)**	13.19	6.19	0.29	-0.46
**Number of types of events**	2.08	0.98	0.67	-0.18
**Frequency of events**	3.49	1.17	-0.13	-0.98

M = Mean; SD = Standard deviation; Skew. = Skewness; Kurt. = Kurtosis.

Reported traumatic events as well as what the participants considered the most distressing events are presented in Table 3. Most of the participants saw dead bodies (n = 119; 89.47%) and thought of this experience as the most distressing (n = 77; 57.46%).

**Table 3:** Traumatic events as reported by the participants.

Event	Reported by the participants (%) n = 133*	Identified as evoking greatest shock, causing trauma, as something they could not forget (%) n = 134**
**1. You were attacked and/ your life was directly threatened.**	15 (11.28%)	11 (8.21%)
**2. You were attacked and/ got injured.**	2 (1.50%)	0 (0%)
**3. Other firefighters were attacked and/ their lives were directly threatened.**	18 (13.53%)	5 (3.73%)
**4. Other firefighters were attacked and/ got injured.**	5 (3.76%)	1 (0.75%)
**5. One of the firefighters was killed.**	10 (7.52%)	10 (7.46%)
**6. You saw dead bodies.**	119 (89.47%)	77 (57.46%)
**7. You saw dead bodies, including children.**	57 (42.86%)	24 (17.91%)
**8. You injured someone.**	5 (3.76%)	2 (1.49%)
**9. You were in a hostile and aggressive crowd.**	26 (19.55%)	4 (2.99%)

* Percentages do not add up because the participants’ task was to tick off all the traumatic events they experienced at work.

**Percentages do not add up because 3 participants chose 2 events as the most distressing.

Taking into consideration the frequency of experiencing a potentially traumatic event (n = 131), the majority of participants reported experiencing them several times (32.06%); 17.56% experienced them between ten and twenty times; and more than that—25.19%. 17.56% experienced a traumatic event once, while only 7.63% did not experience any traumatic event on duty.

As has already been noted, the selected method of computing probable PTSD on the basis of IES-R scores was to possess mean values of all the three sub-scales equal to or higher than 1.5 [61]. This cut-off in the current sample revealed that 46 (34.07%) of the participants suffer from probable PTSD.

The analysis of the correlation between social relations/support, intrusions, hyperarousal, avoidance, the IES-R total score, social status norms, toughness norms, anti-femininity norms, age, relationship length, length of service, number of types of events, and frequency of events (Table 4) revealed a statistically significant mildly negative association between social relations/support and intrusions (-.271), hyperarousal (-.319), the total score of the IES-R scale (-.254) and frequency of events (-.201).

**Table 4:** Correlations between PTSD symptomatology, male role norms, social support and sociodemographic data.

Variables	(1)	(2)	(3)	(4)	(5)	(6)	(7)	(8)	(9)	(10)	(11)	(12).	(13)
**1. SR/S**	1	-.271^A^**	-.319^A^***	-.136^A^	-.254^A^**	-.048^A^	-.115^A^	-.112^A^	-.019^A^	-.014^A^	-.077^A^	-.117^A^	.201^A^*
**2. Intr.**		1	.888^A^***	.828^A^***	.946 ^A^***	.352^A^***	.200^A^*	.102 ^A^	.348^A^***	.364^A^***	.419^A^***	.457^A^***	.231^A^*
**3. Hyp.**			1	.876^A^***	.964^A^***	.313^A^***	.254^A^**	.106 ^A^	.286^A^**	.328^A^**	.412^A^***	.472^A^***	.170^A^
**4. Av.**				1	.944^A^***	.359^A^***	.289^A^***	.153^A^	.300^A^***	.332^A^***	.353^A^***	.490^A^***	.224^A^*
**5. IES-R**					1	.363^A^***	.267 ^A^**	.123 ^A^	.300^A^***	.332^A^***	.406^A^***	.494^A^***	.221^A^*
**6. SSN**						1	.593^A^***	.347^A^***	.205^A^*	.222^A^*	.242^B^*	.382^A^***	.195^A^*
**7. TN**							1	.369^A^***	.060^A^	-.003^A^	.112^A^	.251^A^**	.118^A^
**8. AFN**								1	.036 ^A^	-.131 ^A^	.024 ^A^	.180^A^*	.066^A^
**9. Ag**									1	.787^A^***	.873^A^***	.406^A^ ***	.378^A^***
**10. RL (yrs)**										1	.662^A^***	.251^A^ *	.249^A^***
**11. LoS (yrs)**											1	.448^A^***	.513^A^***
**12. No. ev.**												1	.359^A^***
**13. Freq. ev.**													1

SR/S = Social relations/support; Intr. = Intrusions; Hyp. = Hyperarousal; Av. = Avoidance; IES-R = The IES-R total score; SSN = Social status norms; TN = Toughness norms; AFN = Anti-femininity norms; Ag. = Age; RL (yrs) = Relationship length; LoS (yrs) = Length of service; No. ev. = Number of types of events; Freq. ev. = Frequency of events;

^**A**^Spearman’s rank correlation coefficient,

^B^Pearson’s correlation coefficient,

*p< .05,

**p< .01,

***p< .001.

In the case of intrusions, the variable analysis of correlation revealed a mildly positive association with social status (.352), toughness norms (.200), age (.348), relationship length (.364), length of service (.419), number of types of events (.457) and frequency of events (.231).

The analysis showed statistically significant positive correlations between hyperarousal and social status (.313) and toughness norms (.254), age (.286), relationship length (.328), length of service (.412) and number of types of events (.472).

There were observed mildly positive correlations between the avoidance variable and social status (.359), toughness norms (.289), age (.300), relationship length (.332), length of service (.353), number of types of events (.490) and frequency of events (.224).

In the case of the IES-R total score variable, the analysis revealed statistically significant mildly positive association with social status (.363) and toughness norms (.267), age (.300), relationship length (.332), length of service (.406), number of types of events (.494) and frequency of events (.221).

The next conducted analysis aimed to assess whether firefighters with potential PTSD differed from firefighters who probably did not suffer from PTSD in perceived social support and in the level of compliance with the male role norms.

The Mann-Whitney U test revealed statistically significant differences between the group most likely to suffer from PTSD (whose mean values of the intrusions, hyperarousal and avoidance sub-scale were equal or higher than 1.5—the positive screen for PTSD) and the group without PTSD (where the conditions mentioned above were not met—the negative screen for PTSD) (see Table 5)—the former scored significantly higher in toughness and anti-femininity norms, while the latter scored significantly higher in the relationships/social support. In the case of social status norms, one-way ANOVA revealed that the positive screen for the PTSD group (M = 4.96; SD = 0.89) scored significantly higher (F = 24.149; p< .001; Ƞ^2^_p_ = 0.154) than the negative screen for the PTSD group (M = 4.06; SD = 0.89).

**Table 5:** Results of the Mann–Whitney U test–intergroup differences in perceived social support and male role norms between the groups with the positive screen for PTSD and the negative screen for PTSD.

Variables	Positive screen for PTSD		Negative screen for PTSD		p	Cohen’s Effect size d
	M	SD	M	SD		
**Social relations/support**	3.14	0.66	3.52	0.82	.004	0.511
**Toughness norms**	4.40	0.98	3.94	0.79	.002	0.517
**Anti-femininity norms**	4.01	1.09	3.47	1.14	.015	0.484

Next, a series of univariate regressions (Table 6) was conducted to identify possible independent predictors of PTSD symptomatology among male role norms, social relations/support, and sociodemographic and trauma-related data. The analysis revealed that age (p< .001), length of service (p< .001), relationship length (p = .003), number of types of events (p< .001), frequency of events (p = .019), social status (p< .001) and toughness norms (p = .008) are independent positive predictors of PTSD symptomatology, while education (p = .016) and social relations/support (p< .001) are negative predictors.

**Table 6:** Results of the series of univariate regressions with the IES-R total score as a dependent variable and the series of logistic regressions with probable PTSD and no PTSD as a dependent variable and male role norms, social relations/ support, and sociodemographic and trauma-related data as independent predictors in both cases.

Predictors	β	SEE β	T	R^2^	Adj. R^2^	F	df	p	OR (95%CI)**	p
**Ag.**	.32	0.08	3.82	.11	.10	14.58	1.124	< .001	1.16 (1.08–1.24)	< .001
Edu.*	-.21	0.08	-2.44	.04	.04	5.97	1.113	.016	0.37 (0.16–0.85)	.018
**LoS (yrs)**	.43	0.08	5.45	.19	.18	29.70	1.130	< .001	1.23 (1.13–1.33)	< .001
**RL (yrs)**	.30	0.10	3.04	.09	.08	9.23	1.930	.003	1.13 (1.05–1.22)	.001
**No. ev.**	.47	0.08	6.09	.22	.22	37.14	1.130	< .001	3.79 (2.18–6.61)	< .001
**Freq. ev.**	.21	0.09	2.38	.04	.03	5.68	1.129	.019	1.40 (1.03–1.90)	.031
**SR/S**	-.31	0.08	-3.76	.10	.09	14.12	1.131	< .001	0.43 (0.25–0.74)	.002
**SSN**	.37	0.08	4.58	.14	.13	20.99	1.133	< .001	2.47 (1.61–3.80)	< .001
**TN**	.23	0.08	2.67	.05	.04	7.14	1.132	.008	1.84 (1.21–2.79)	.004

Ag. = Age; Edu. = Education; LoS (yrs) = Length of service; RL (yrs) = Relationship length; No. ev. = Number of types of events; Freq. ev. = Frequency of events; SR/S = Social relations/support; SSN = Social status norms; TN = Toughness norms;

*dummy-coded: 0—up to secondary education, 1—higher education,

**dichotomized dependent variable–probable PTSD (1) and no PTSD (0).

Then a series of logistic regressions (Table 6) was conducted with the above-mentioned independent variables and the dichotomized dependent variable–probable PTSD (1) and no PTSD (0), based on the IES-R cut-off points used by Juczyński & Ogińska-Bulik [61]. The analysis revealed that age, length of service, relationship length, number of types of events, frequency of events, social status and toughness norms significantly increase the odds of developing PTSD, while education and social relations/support decrease the odds of developing it.

A stepwise backward linear regression (Table 7) (the full table of the regression is included in S1 Table) was then conducted with the IES-R total score as a dependent variable and age, education, length of service, relationship length, number of types of events, frequency of events, social relations/support, social status and toughness norms as independent variables. The final model included social status norms (p = .002) and number of types of events (p< .001) as statistically significant predictors of PTSD symptomatology, accounting for 28% of its variance.

**Table 7:** A summary of the results of the backward stepwise linear regression with the IES-R total score as a dependent variable, and male role norms, social relations/support, and sociodemographic and trauma-related data as independent variables.

Predictors	β	SEE β	t	p	df	R^2^	Adj. R^2^	F
**No ev.**	.38	0.10	3.97	< .001	2.83	.30	.28	17.40
**SSN**	.31	0.10	3.22	.002				

No. ev. = Number of types of events; SSN = Social status norms.

## Discussion

The primary aim of the study was to explore potential associations between compliance with male role norms and PTSD symptomatology in a sample of firefighters while controlling the demographic covariates. Statistical analyses revealed significant positive associations between IES-R total score, its sub-scales of intrusions, hyperarousal and avoidance, social status and toughness norms, age, relationship length, length of service, number of types of events and frequency of events; and a negative association in the case of social relations/support.

In traditionally masculine societies, such as Poland [67], the positive self-concept may be strongly associated with conforming to cultural standards/norms of masculinity [68]. Sanctioning hegemonic masculinity [69,70] makes it necessary to meet the criteria of masculinity and constantly confirm one’s role [71]. These results might be interpreted in the following way: male role norms may be associated with the reinforcement of certain beliefs (e.g. concerning emotions in general, the ways of (not) expressing them, about the irrelevance of safety and health in general) [72–74] that reduce one’s ability to adaptively process and regulate emotions in the face of potentially traumatic events. This result seems to be in accord with the so-called “Male Normative Alexithymia Hypothesis” [75], according to which lifelong conformity to the masculine norms pertaining to restrictive emotionality reduces the chances of obtaining abilities to properly identify and describe emotional states. Such an interpretation of the observed associations seems to be supported by the etiology of PTSD proposed by Foa & Kozak [76] and Berke, Reidy & Zeichner’s model of masculinity and men’s psychopathology [47]. This assumption can be further reinforced by the observed positive association between avoidance symptoms of PTSD and traditional male role norms (except for anti-femininity norms). Similar results were found in the study conducted by Elder and associates [46]. It is worth mentioning that no significant association has been observed between anti-femininity norms and symptoms of PTSD in the current study. This might mean that complying with anti-femininity norms might not be related to increased PTSD symptomatology—so only social status and toughness norms seem to reinforce maladaptive enough strategies/behaviors that significantly contribute to the development of PTSD symptoms in the face of potentially traumatic stimuli.

The intergroup differences analysis showed that the group consisting of firefighters with probable PTSD significantly differed from the group without PTSD in compliance with male role norms and in relationships/social support. Individuals suffering from PTSD may experience shame and the sense of guilt [77]; thus to investigate the relation between shame and PTSD [78], it is worth interpreting the results employing Nathanson’s Compass Shame model [79]. Firefighters, who conform to a great extent to the male role norms, may experience shame as a reaction to trauma (where being affected by the trauma might be seen by one as being vulnerable, weak, thus not conforming to the male role norms), which is in turn associated with an increased tendency toward negative self-evaluation [80] and self-critical thinking [81], which may result in employing maladaptive styles of coping with shame. Taking the above into consideration, they may resort to aggression aimed at others (perceived as hypermasculine behavior) [82] to restore their self-worth [83–85], though at the same time this might decrease the levels of available social support and the sense of belongingness. They may also employ avoidance strategies e.g., firefighters suffering from PTSD may avoid social contacts (diminishing the chance of perceiving social support or obtaining it), and have a thwarted sense of belongingness [55]. For fear of being rejected, they may isolate themselves from others. These issues might result in employing other maladaptive strategies of emotion regulation, such as substance abuse [79,86,87], e.g., alcohol abuse [88–90]. These strategies may also be reinforced by other co-occurring mental health conditions, such as depression [91]. The other way to interpret these results might be similar to those found in the study conducted by Elder and associates [46], where traditional masculinity norms (their behavioral scripts) are associated with the reinforcement of the avoidance symptoms of PTSD or even conforming to the male norms as a symptom of avoidance. More strict endorsement of masculine norms among firefighters with probable PTSD might also be interpreted as a coping strategy—acting “manly” to avoid/cope with shame [77] and potential future trauma/stressful events.

The need for support corresponds with gender stereotypes: according to the masculinity stereotype, men facing difficulties seek seclusion and do not show the need for support [70]. Many firefighters consider the expression of emotions (e.g. shame or sadness) as feminine and thus unacceptable [82], which, as a consequence, may lead to extreme callousness toward other people [74]. Besides, the necessity to communicate the need for support would involve the disclosure of one’s weakness which could cause the loss of self-esteem and respect in other people’s eyes [72].

Positive PTSD screens were associated with social status norms, toughness norms, older age, increased length of service, increased relationship length, increased number of types of events, increased frequency of events, decreased social relations/support and lower education.

The results of the study might imply that time spent in service and the need to achieve high status (which might result in gender strain) are positively associated with the development of PTSD symptomatology [38]. This might be interpreted in the following way: strict compliance with social status and toughness norms and the needs associated with them might be positively associated with gender strain [38], thus with maladaptive ways of cognitive and emotional processing in the face of potentially traumatic events, and with the development of PTSD symptomatology [76]; while the longer time of service might be positively associated with the increased probability of experiencing a potentially traumatic event. The increased number of types of events, as well as their increased frequency, might be associated with “cognitive overload”—a decreased ability to process potentially traumatic stimuli and thus with the increased probability of PTSD. Older age might pose a burden in that process in way that it involves more instances and longer time of exposition to potentially traumatic events, which is positively associated with the increased probability of PTSD. Increased relationship length was associated with probable PTSD, and the results are somehow contrary to the findings of Alghamdi and associates [92]. The results of the current study might suggest that maintaining the relationship might be associated with thinning of the resources (where some might be redistributed to cope with potential relationship conflicts, whose chances might increase with the relationship length) that could be used in coping with potentially traumatic stimuli.

In the case of the perceived social support, the results are similar to the results obtained by Meyer and associates [35], Mitani [34] and Shakespeare-Finch, Rees & Armstrong [33]. Higher education was found to be negatively associated with the risk of development symptoms of PTSD; similar results were found in the study by Alghamdi and associates [92]. In both cases, this might be associated with acquiring more resources to cope with potentially traumatic stimuli. The higher levels of perceived social support by trauma-exposed firefighters might be associated to more adaptive coping strategies that were promoted by the support givers [55].

The analysis of the obtained data in the backward stepwise regression indicated that compliance with male role norms pertaining to social status and number of types of potentially traumatic events is more strongly associated with the development of PTSD symptomatology than the rest of the variables mentioned in the univariate analyses.

As far as toughness norms are concerned, the results of the multivariate analysis differ from the study conducted by Jakupcak and associates [45] on a sample of Afghanistan veterans, where these norms were a significant predictor of the development of PTSD and depression, whereas in the current study such an instance has not been observed. This may yield a few implications: toughness norms are not as strongly associated with firefighters’ overall functioning as in the case of war veterans; this may be due to cultural differences (assuming that in Polish culture status matters more than toughness in masculine ideology); or the explanation might lie in the relatively small sample size, which made it impossible to fully show potential tendencies in the group.

The masculinity stereotype refers to a professional role: men may choose a profession conforming to the stereotype [93,94]. The man should achieve professional success [71], while a strong need for success is often related to having a traditional attitude to gender roles [95]. Firefighting is not only a highly-respected profession, but it also requires presenting stereotypically masculine traits, such as courage, and physical and psychic strength [96,97]. Thus, performing the profession may be associated with the reinforcement of the expectations of masculine behaviors [98–100], which may make it difficult for firefighters to create satisfactory relations [101].

### Limitations

Having analyzed the presented results, we have to take into account several issues and limitations of our study. First, the chain referral method and convenience sampling—while it is a convenient way of acquiring study groups, it might have a potentially negative impact on the data elicited from those participants who are eager to participate and reveal personal and potentially painful information about their experiences [75]. The tendency to avoid things that remind them of traumatic events among those with PTSD might severely reduce their participation in the study, which might result in underestimated rates of clinically significant PTSD symptomatology and its associations as presented in the current study. This might also be caused by the relatively small sample size and information omitted by the participants (the report bias).

Second, another limitation that should be considered is the recall bias. Due to long years of service, the participants could forget much information concerning the frequency of potentially traumatic events could be forgotten by the participants, which could result in the lower reliability of the presented results.

Third, given the Polish traditionalist approach to manhood/masculinity, the participants might overestimate their compliance with the male role norms [67,68]. For the very same reason, the intensity of PTSD symptomatology might be underestimated—the participants might consider it “unmanly” to admit to suffering from it—and the reported perceived social support and might deviate from reality, as the participants might not want to disclose that they felt lonely.

The fourth limitation of the current study is its cross-sectional nature, which makes it impossible to assess the relations between PTSD symptomatology, male role norms, social relations/support and sociodemographic data in the long run.

Fifth, the sample was limited to firefighters working in big urban centers; thus the results of the study might probably apply only to them, but not to the firefighters working in small towns and rural areas.

Sixth, the study did not take into consideration other potential mental health issues of the respondents, drug and alcohol use and other adverse experiences, e.g., those from the childhood. They might potentially increase the reported levels of PTSD symptomatology [11,26–29].

## Conclusions

The aim of our study was to explore potential associations between compliance with the traditional male role norms and PTSD symptoms among Polish firefighters. Having analyzed the results of our research, we have come to the following conclusions:

1. Social status and toughness norms are significantly associated with the increased risk of the development of PTSD symptoms.

Firefighting may be associated with reinforcement of stereotypically masculine behaviors, which in turn are associated with a decreased ability to cope with PTSD, and favor maladaptive behaviors. Besides, the identification with a professional role may be associated with difficulties for firefighters to get support from important others, and with the increase of PTSD symptoms. In the case of social status norms, the analysis showed that upholding strict beliefs about social status is positively associated with the increased risk of developing symptoms of PTSD, which accords with the theories formulated by Pleck [38] and O’Neill [39].

2. Over 34% of the sample of Polish firefighters reported clinically significant [61] symptomatology placing them in the group of probably suffering from PTSD.

This figure falls in the middle of the reported rates of PTSD in firefighters mentioned earlier in this paper [3–11]. Yet in comparison to other studies conducted on Polish firefighters, the rate of prevalence of PTSD seems to be similar to that presented in the study by Witt, Stelcer & Czarnecka-Iwańczuk [102], but it is more than twice as high as that reported by Ogińska-Bulik & Langer [26].

3. The prevalence of probable PTSD among the studied group still highlights the problem of the population underserved by mental health care services.

The results might yield important clinical implications—that psychological intervention ought to assess and target firefighters’ maladaptive beliefs concerning masculinity, which might be especially beneficial in the case of PTSD treatment. Firefighters with the positive screen for PTSD may be reluctant to express their emotions and face problems with emotion regulation [103,104]; their problems in this respect may be intensified by their willingness to live up to the social expectations concerning the performance of the traditional male role, that is why it is vital to take into consideration their masculinity in the treatment [47,82].

## Supporting information

S1 Table Results of the backward stepwise regression. (DOCX)
