# Supplementary material for: The impact of the traditional male role norms on the posttraumatic stress disorder among Polish male firefighters
Source: PLoS One. 2021 Oct 27;16(10):e0259025. doi: 10.1371/journal.pone.0259025 (PMC8550442; doi:10.1371/journal.pone.0259025)
Supplement: S1 Table — (DOCX) [file pone.0259025.s001.docx]

## S1 Table. Results of the backward stepwise regression with the IES-R total score as a dependent variable, and male role norms, social relations/ support, and sociodemographic and trauma-related data as independent variables.

| **Step** | **Predictors** | **β** | **SEE β** | **Β** | **SEE B** | **t** | **p** | **df** | **R^2^** | **Adj. R^2^** | **F** |
| --- | --- | --- | --- | --- | --- | --- | --- | --- | --- | --- | --- |
| **Step 0** | Ag. | .19 | 0.01 | .03 | 0.49 | 0.62 | .624 | 9.760 | .42 | .35 | 6.15 |
|  | Edu.* | .09 | -0.16 | .19 | -0.85 | 0.40 | .400 |  |  |  |  |
|  | LoS (yrs) | .20 | 0.02 | .03 | 0.48 | 0.63 | .630 |  |  |  |  |
|  | RL (yrs) | .14 | <0.01 | .02 | 0.04 | 0.97 | .967 |  |  |  |  |
|  | No.ev. | .10 | 0.26 | .10 | 2.60 | 0.01 | .011 |  |  |  |  |
|  | Freq. ev. | .12 | 0.13 | .10 | 1.25 | 0.22 | .216 |  |  |  |  |
|  | SR/S | .10 | -0.31 | .13 | -2.41 | 0.02 | .019 |  |  |  |  |
|  | SSN | .10 | 0.24 | .10 | 2.32 | 0.02 | .023 |  |  |  |  |
|  | TN | .10 | 0.01 | .12 | 0.08 | 0.94 | .937 |  |  |  |  |
| **Step 1** | Ag. | .10 | 0.17 | .01 | 0.03 | 0.57 | .569 | 8.77 | .42 | .36 | 7.01 |
|  | Edu.* | -.08 | 0.09 | -.17 | 0.19 | -0.86 | .392 |  |  |  |  |
|  | LoS (yrs) | .10 | 0.20 | .02 | 0.03 | 0.49 | .622 |  |  |  |  |
|  | No. ev. | .26 | 0.10 | .26 | 0.10 | 2.62 | .010 |  |  |  |  |
|  | Freq. ev. | .15 | 0.12 | .13 | 0.10 | 1.28 | .206 |  |  |  |  |
|  | SR/S | -.24 | 0.10 | -.31 | 0.13 | -2.42 | .018 |  |  |  |  |
|  | SSN | .24 | 0.10 | .24 | 0.10 | 2.34 | .022 |  |  |  |  |
|  | TN | 01 | 0.10 | .01 | 0.11 | 0.08 | .935 |  |  |  |  |
| **Step 2** | Ag. | .10 | 0.17 | .01 | 0.03 | 0.57 | .569 | 7.78 | .42 | .37 | 8.11 |
|  | Edu.* | -.08 | 0.09 | -.17 | 0.19 | -0.89 | .374 |  |  |  |  |
|  | LoS (yrs) | .10 | 0.20 | .02 | 0.03 | 0.49 | .622 |  |  |  |  |
|  | No. ev. | .26 | 0.10 | .26 | 0.10 | 2.65 | .010 |  |  |  |  |
|  | Freq. ev. | .16 | 0.12 | .13 | 0.10 | 1.29 | .199 |  |  |  |  |
|  | SR/S | -.25 | 0.10 | -.31 | 0.12 | -2.49 | .015 |  |  |  |  |
|  | SSN | .25 | 0.09 | .25 | 0.09 | 2.70 | .008 |  |  |  |  |
| **Step 3** | Ag. | .17 | 0.09 | .02 | 0.01 | 1.80 | .075 | 6.79 | .42 | .38 | 9.51 |
|  | Edu.* | -.08 | 0.09 | -.17 | 0.19 | -0.94 | .351 |  |  |  |  |
|  | No. ev. | .27 | 0.10 | .27 | 0.10 | 2.80 | .006 |  |  |  |  |
|  | Freq. ev. | .19 | 0.10 | .16 | 0.08 | 1.92 | .059 |  |  |  |  |
|  | SR/S | -.26 | 0.09 | -.33 | 0.12 | -2.81 | .006 |  |  |  |  |
|  | SSN | .25 | 0.09 | .25 | 0.09 | 2.72 | .008 |  |  |  |  |
| **Step 4** | Ag. | .18 | 0.09 | .03 | 0.01 | 1.92 | .059 | 5.80 | .41 | .38 | 11.25 |
|  | No. ev. | .28 | 0.10 | .28 | 0.10 | 2.90 | .005 |  |  |  |  |
|  | Freq. ev. | .19 | 0.10 | .16 | 0.08 | 1.96 | .054 |  |  |  |  |
|  | SR/S | -.28 | 0.09 | -.35 | 0.11 | -3.10 | .003 |  |  |  |  |
|  | SSN | .25 | 0.09 | .25 | 0.09 | 2.83 | .006 |  |  |  |  |
| **Step 5** | No. ev. | .31 | 0.10 | .31 | 0.10 | 3.22 | .002 | 4.81 | .39 | .36 | 12.73 |
|  | Freq. ev. | .23 | 0.10 | .19 | 0.08 | 2.29 | .025 |  |  |  |  |
|  | SR/S | -.29 | 0.09 | -.37 | 0.12 | -3.17 | .002 |  |  |  |  |
|  | SSN | .28 | 0.09 | .28 | 0.09 | 3.14 | .002 |  |  |  |  |
| **Step 6** | No. ev. | .39 | 0.09 | .39 | 0.09 | 4.23 | <.001 | 3.82 | .35 | .32 | 14.49 |
|  | SR/S | -.23 | 0.09 | -.29 | 0.11 | -2.53 | .013 |  |  |  |  |
|  | SSN | .29 | 0.09 | .29 | 0.09 | 3.18 | .002 |  |  |  |  |
| **Step 7** | No. ev. | .38 | 0.10 | .38 | 0,10 | 3.97 | <.001 | 2.83 | .30 | .28 | 17.40 |
|  | SSN | .31 | 0.10 | .31 | 0.09 | 3.22 | .002 |  |  |  |  |

Ag.= Age; Edu.= Education; LoS (yrs)= Length of service; RL (yrs)= Relationship length; No. ev.= Number of types of events; Freq. ev.= Frequency of events; SR/S= Social relations/ support; SSN= Social status norms; TN= Toughness norms; *dummy-coded: 0 — up to secondary education, 1 — higher education.
